# Correlation between anemia-related red blood cell parameters and pulmonary function severity in patients with AECOPD

**DOI:** 10.3389/fmed.2026.1773007

**Published:** 2026-03-04

**Authors:** Hui Dou, Jichen Qiao, Hailian Zhang, Dabang Cai, Cheng Meng, Xin Wei, Linghua Cui, Shumei Shao, Lei Lv

**Affiliations:** 1 Department of Clinical Laboratory Medicine, Suzhou First People’s Hospital, Suzhou, Anhui, China; 2 Department of Basic Medicine, Fuzhou Medical College, Fuzhou, Jiangxi, China; 3 Department of Respiratory Medicine, Suzhou First People’s Hospital, Suzhou, Anhui, China

**Keywords:** anemia, chronic obstructive pulmonary disease, lung function, mean corpuscular hemoglobi, prognosis, red cell distribution width

## Abstract

**Aim:**

This study aimed to investigate the prevalence of anemia and the correlation between specific red blood cell (RBC) parameters—particularly red cell distribution width (RDW) and mean corpuscular hemoglobin (MCH)—and pulmonary function severity in patients with acute exacerbation of chronic obstructive pulmonary disease (AECOPD).

**Methods:**

A retrospective, single-center cohort study was conducted involving 120 hospitalized AECOPD patients. Participants were stratified by lung function grade (GOLD 1–4) and anemia status. Data on demographics, RBC parameters (Hb, RDW, MCH, MCV), and clinical outcomes were collected. Statistical analyses included correlation tests, ROC curve analysis, multivariate logistic regression, and Kaplan-Meier survival analysis.

**Results:**

The overall anemia prevalence was 26.7%, increasing significantly with lung function severity, exceeding 50% in GOLD grade IV. RDW exhibited a significant negative correlation with FEV1%pred (*r* = –0.418, *P* < 0.001), while MCH showed a positive correlation (*r* = 0.391, *P* < 0.001). Multivariate analysis identified decreased Hb and elevated RDW as independent factors associated with severe lung impairment (Grades III–IV). The anemia group had significantly longer hospital stays, and patients with high RDW levels demonstrated significantly reduced survival.

**Conclusion:**

Anemia is highly prevalent in AECOPD and is closely but moderately associated with disease severity. RDW and MCH are indicators that show independent associations with lung function impairment and selected clinical outcomes in this cohort, but these relationships cannot be interpreted as causal because of the observational study design. Integrating these readily available RBC parameters into routine assessment could help enhance risk stratification when interpreted alongside established clinical and physiological markers.

## 1 Introduction

Chronic Obstructive Pulmonary Disease (COPD), a prevalent chronic condition characterized by persistent airflow limitation, represents a major global public health challenge due to its high prevalence, significant disability burden, and elevated mortality rates. The disease not only affects the lungs, leading to chronic cough, sputum production, and progressively worsening dyspnea, but also triggers various notable extrapulmonary effects and systemic comorbidities (1), contributing to its complex clinical profile. Among these, Acute Exacerbation of COPD (AECOPD) is a critical event in the disease trajectory, indicating a sudden deterioration of symptoms. It not only accelerates the decline of lung function but also stands as the primary factor driving increased rehospitalization risks and reduced survival (2).

Among the numerous systemic comorbidities associated with AECOPD, anemia—a frequently underestimated complication—has gained increasing clinical recognition in recent years (3). Anemia, fundamentally a pathological condition marked by diminished oxygen-carrying capacity of the blood, imposes a substantial global burden according to World Health Organization data (4). When anemia coexists with AECOPD, the two conditions can interact synergistically in detrimental ways. On one hand, the inherent chronic hypoxic state and systemic inflammatory milieu in AECOPD (5) are thought to contribute to anemia as a systemic comorbidity by suppressing erythropoietin activity and disrupting iron metabolism homeostasis, representing a pattern commonly classified as “anemia of chronic disease” (6). On the other hand, the presence of anemia further impairs tissue oxygen delivery, thereby aggravating pre-existing tissue hypoxia in AECOPD patients and creating a vicious cycle that unnecessarily increases respiratory load (7). Substantial clinical evidence indicates that AECOPD patients with comorbid anemia experience significantly prolonged hospital stays, consume more healthcare resources, and face a multiplied risk of long-term mortality (8). Therefore, early identification of anemia in AECOPD patients and timely optimization of underlying COPD management and comorbid conditions, together with systematic evaluation and treatment of reversible causes of anemia (such as iron deficiency and nutritional factors), may help reduce symptom burden, identify high-risk patients and potentially mitigate socioeconomic impact.

In clinical practice, complete blood count testing serves as the most direct and routine method for screening and diagnosing anemia. However, conventional diagnostic approaches often focus solely on whether hemoglobin (Hb) levels fall below a cutoff value, which inevitably limits the exploration of deeper insights into anemia. In reality, automated hematology analyzers provide a range of valuable red blood cell (RBC) parameters that collectively constitute a distinctive “identity fingerprint” of anemia. For instance, red cell distribution width (RDW), a key indicator reflecting heterogeneity in red blood cell volume, typically increases in response to impaired erythropoiesis or ineffective red cell production, and is closely associated with sustained inflammatory stress and oxidative damage in the body (9). Accumulating evidence indicates that elevated RDW is associated with more severe airflow limitation, higher risk of acute exacerbations, hospital readmission and increased mortality in patients with COPD and AECOPD (10, 11). In addition, RDW has been reported to be significantly increased in patients with obstructive sleep apnea syndrome (OSAS) and in overlap syndrome (COPD with OSAS), and higher RDW values correlate with apnea–hypopnea index and surrogate cardiovascular risk markers, further supporting its role as a simple, inexpensive marker of systemic hypoxia-inflammation burden (12, 13).

Meanwhile, mean corpuscular hemoglobin (MCH) and mean corpuscular volume (MCV) are essential for the initial differentiation of anemia etiology (14) (e.g., microcytic hypochromic, normocytic, or macrocytic anemia). In recent years, cutting-edge research has begun to explore the prognostic value of these parameters in the field of COPD. Some scholars have identified RDW as an independent predictor of exacerbation risk in COPD patients (15), while other studies have reported that low MCH levels are closely linked to adverse outcomes in AECOPD patients (16).

Although existing studies have revealed associations between anemia and overall COPD prognosis, a key scientific question remains unresolved: within the specific AECOPD population, do these refined RBC parameters (such as RDW and MCH) exhibit regular, quantifiable changes as the severity of lung function impairment increases? Currently, there is a lack of comprehensive literature reporting systematic comparisons (17) and correlation analyses of RDW, MCH, and other indicators across AECOPD patients with different lung function grades (GOLD 1–4). This knowledge gap somewhat hinders clinicians from utilizing readily available complete blood count reports to more accurately assess disease severity in AECOPD patients (18).

Based on the current research landscape, this study leverages in-depth collaboration between hospital laboratory medicine and respiratory departments, capitalizing on the strengths of multidisciplinary cooperation. We aim to conduct a retrospective analysis of 120 AECOPD patients admitted in the past 2 years to systematically investigate the following core aspects: first, to clarify the distribution characteristics of anemia and different types of anemia in the AECOPD population; second, to dynamically observe the trajectories of key RBC parameters such as RDW and MCH across patients with varying severities of lung function impairment; and finally, to evaluate the correlation of these parameters with FEV1% predicted values and their predictive efficacy for severe AECOPD. This study aims to provide a novel, economical, and efficient laboratory reference dimension for the clinical assessment of AECOPD (19), thereby contributing to individualized patient management and prognostic evaluation.

## 2 Materials and methods

### 2.1 Study subjects and design

This study employed a retrospective, single-center, observational cohort design. The study subjects were consecutively enrolled from patients hospitalized for AECOPD in the Department of Respiratory and Critical Care Medicine of our hospital between January 1, 2023, and December 31, 2024. The study protocol was approved by the Ethics Review Committee of our hospital. Given the retrospective nature of the research, the Ethics Committee waived the requirement for obtaining informed consent from patients; however, all patient information was anonymized after collection to strictly protect patient privacy.

### 2.2 Inclusion and exclusion criteria

To ensure the homogeneity of the study population and the reliability of the data, clear inclusion and exclusion criteria were established.

Inclusion criteria comprised: (1) Meeting the diagnostic criteria for AECOPD as outlined in the “Guidelines for the Diagnosis and Treatment of Chronic Obstructive Pulmonary Disease (2021 Revision),” specifically the presence of sustained worsening of respiratory symptoms (e.g., intensified cough, increased sputum volume, purulent sputum, aggravated dyspnea) beyond normal daily variation, necessitating a change in routine medication; (2) Completion of pulmonary function tests during hospitalization after the treating respiratory physician judged that the patient had achieved clinical stabilization, with FEV1/FVC < 0.7, thereby confirming persistent airflow limitation. Clinical stabilization in this study referred to improvement in dyspnea, absence of acute respiratory distress, stable vital signs (temperature, heart rate, blood pressure and respiratory rate within acceptable ranges for the individual patient), and no need for escalation of oxygen therapy, non-invasive ventilation or vasopressor support; (3) Completion of a complete blood count test within 24 h of admission, with relevant data being complete and accessible; (4) Age ≥ 40 years.

Exclusion criteria aimed to eliminate other confounding factors that could significantly affect anemia status or RBC parameters, specifically including: (1) Comorbid severe anemia due to other known causes, such as active gastrointestinal bleeding, gynecological blood loss, definitively diagnosed hematologic malignancies (e.g., leukemia, lymphoma, multiple myeloma), myelodysplastic syndromes, hemolytic anemia, etc.; (2) Comorbid severe liver dysfunction (Child-Pugh Class C) or severe renal insufficiency (chronic kidney disease stages 4–5 or receiving dialysis treatment), as these conditions themselves can lead to renal anemia or anemia of liver disease; (3) History of blood transfusion within the past 3 months, as transfusion can transiently interfere with authentic RBC parameters; (4) Severe lack of clinical data, preventing accurate grouping or analysis; (5) Comorbid other chronic respiratory diseases affecting lung function, such as bronchiectasis, pulmonary fibrosis, destroyed lung due to tuberculosis, severe thoracic deformity, etc.

Through strict screening using the above criteria, 120 AECOPD patients were ultimately included in this analysis from an initial screening of 156 patients.

### 2.3 Research methods and grouping

#### 2.3.1 Data collection

Two uniformly trained researchers independently extracted data from the hospital’s electronic medical record system using a self-designed “Case Information Collection Form” and performed cross-verification to ensure data accuracy. The collected content included:

1. Demographic data: age, sex, height, weight (used to calculate Body Mass Index, BMI).
2. Clinical data: admission date, discharge date (used to calculate length of hospital stay), comorbidity status (focusing on hypoalbuminemia, defined as serum albumin < 35 g/L).
3. Laboratory test data: Collected from the first venous blood test after admission. Complete blood count data were obtained using Sysmex XN series automated hematology analyzers, focusing on: RBC count, Hb, RDW, MCV, and MCH. Serum albumin, C-reactive protein, and other indicators were also recorded.
4. Pulmonary function data: Measured using a German Jaeger MasterScreen pulmonary function tester, recording the post-bronchodilator forced expiratory volume in 1 s as a percentage of the predicted value (FEV1% predicted), which served as the basis for lung function grading.
5. Prognosis follow-up data: Collected through a combination of electronic medical record review and telephone follow-up to determine the survival status of patients after discharge. The follow-up cutoff date was January 31, 2025. The primary endpoint was defined as all-cause mortality.

#### 2.3.2 Grouping criteria

This study utilized two core grouping methods for stratified analysis of patients:

Based on lung function grading: According to the Global Initiative for Chronic Obstructive Lung Disease 2024 report criteria, patients were divided into 4 grades based on FEV1% predicted: Grade I (mild, FEV1% ≥ 80%), Grade II (moderate, 50% ≤ FEV1% < 80%), Grade III (severe, 30% ≤ FEV1% < 50%), Grade IV (very severe, FEV1% < 30%).

Based on anemia diagnosis: Referring to World Health Organization diagnostic criteria for anemia, defined as Hb < 130 g/L for males residing at sea level, and < 120 g/L for non-pregnant females. Accordingly, patients were categorized into an “Anemia group” and a “Non-anemia group.” These fixed cut-off values were applied irrespective of smoking status; in heavy smokers with secondary polycythemia this definition may underestimate the true prevalence of anemia, and the reported anemia rates should therefore be interpreted as conservative estimates. Furthermore, for deeper analysis, we preliminarily classified patients in the anemia group based on MCV into morphological types: MCV < 80 fL as microcytic anemia, 80 fL ≤ MCV ≤ 100 fL as normocytic anemia, MCV > 100 fL as macrocytic anemia.

### 2.4 Observation indicators

The primary observation indicators in this study included:

1. Baseline characteristics: age, sex, BMI, lung function grade.
2. Core RBC parameters: RBC count, Hb, red cell distribution width, MCV, and MCH.
3. Clinical outcome indicators: length of hospital stay, all-cause mortality.

### 2.5 Statistical analysis

All data were statistically analyzed using IBM SPSS Statistics version 26.0 software. Measurement data were first tested for normality (Shapiro-Wilk test). Data conforming to a normal distribution are presented as mean ± standard deviation, and comparisons between groups were performed using one-way analysis of variance; if overall comparisons were statistically significant, the LSD-t test was used for *post-hoc* pairwise comparisons. Data not conforming to a normal distribution are presented as median (interquartile range), and group comparisons were performed using the Kruskal-Wallis H test. Count data are presented as number (percentage), and group comparisons were performed using the chi-square test or Fisher’s exact test (when the number of cells with an expected count < 5 exceeded 20%).

Pearson or Spearman correlation analysis was used to explore the relationships between RBC parameters and FEV1% predicted values. Receiver operating characteristic curve analysis was used to assess the diagnostic efficacy of RDW, MCH, and other indicators for predicting severe AECOPD (i.e., the combined Grade III-IV group), and the area under the curve, optimal cutoff value, sensitivity, and specificity were calculated.

To investigate independent factors influencing the severity of lung function in AECOPD patients, with lung function grade (defining Grades I–II as 0 and Grades III–IV as 1) as the dependent variable, variables with *P* < 0.1 in univariate analysis and clinically significant variables (age, BMI, Hb, RDW, MCH) were included in a multivariate binary logistic regression model (enter method), calculating odds ratios and their 95% confidence intervals. Given the limited number of patients with Grades III–IV impairment and outcome events, the number of covariates in the model was deliberately restricted to avoid overfitting; consequently, other potential confounders such as detailed smoking exposure, prior exacerbation history, comorbid cardiovascular disease and systemic corticosteroid use could not be fully adjusted for, and the multivariable findings should be regarded as exploratory and subject to residual confounding.

For survival analysis, patients were divided into high RDW and low RDW groups based on the median RDW value of the entire cohort (or the optimal prognostic cutoff value determined by ROC curve analysis). Survival curves were plotted using the Kaplan-Meier method, and differences in survival rates between the two groups were compared using the Log-rank test.

All statistical tests were two-sided, and a *P* < 0.05 was considered statistically significant.

## 3 Results

### 3.1 Patient baseline characteristics and anemia prevalence

Among the 120 AECOPD patients ultimately included in this analysis, 82 (68.3%) were male and 38 (31.7%) were female. Grading based on pulmonary function test results showed 22 patients (18.3%) in Grade I (mild), 45 (37.5%) in Grade II (moderate), 38 (31.7%) in Grade III (severe), and 15 (12.5%) in Grade IV (very severe). The distribution was predominantly moderate to severe, consistent with the typical profile of hospitalized AECOPD patients.

Among all patients, 32 met the diagnostic criteria for anemia, resulting in an overall anemia prevalence of 26.7%. A highly significant trend was observed, where anemia prevalence closely correlated with the severity of lung function impairment (*P* < 0.001). As shown in Table 1, from Grade I to Grade IV, anemia prevalence rates were 9.1% (2/22), 17.8% (8/45), 36.8% (14/38), and 53.3% (8/15), respectively. This indicates that over half of the patients with very severe AECOPD had comorbid anemia. Furthermore, as the lung function grade increased, the mean age of patients significantly rose (*P* < 0.001), while body mass index (BMI) significantly declined (*P* < 0.001), suggesting that advanced age and malnutrition might be common clinical features in severe AECOPD patients and are intrinsically linked to the occurrence of anemia. There was no significant difference in gender distribution across the different lung function grade groups (*P* > 0.05).

**TABLE 1:** Comparison of baseline characteristics and anemia prevalence in AECOPD patients by pulmonary function grade (*n* = 120).

Parameter	Total (*n* = 120)	Grade I (*n* = 22)	Grade II (*n* = 45)	Grade III (*n* = 38)	Grade IV (*n* = 15)	*P*-value
Age (years, $\bar{x}$ ± s)	69.2 ± 8.9	66.5 ± 7.8	68.8 ± 8.5	72.1 ± 9.2	75.4 ± 9.8	**< 0.001**
Male [n(%)]	82 (68.3%)	16 (72.7%)	31 (68.9%)	25 (65.8%)	10 (66.7%)	0.921
BMI (kg/m^2^, $\bar{x}$ ± s)	21.8 ± 3.4	23.2 ± 2.9	21.9 ± 3.1	20.1 ± 3.3	18.6 ± 3.6	**< 0.001**
Anemia [n(%)]	32 (26.7%)	2 (9.1%)	8 (17.8%)	14 (36.8%)	8 (53.3%)	**< 0.001**

Data are presented as mean ± standard deviation or number (percentage). *P*-value is for comparison among different pulmonary function grades. Bold values indicate statistically significant differences (*P* < 0.05).

### 3.2 Comparison of clinical characteristics between anemia and non-anemia groups

**FIGURE 1:**
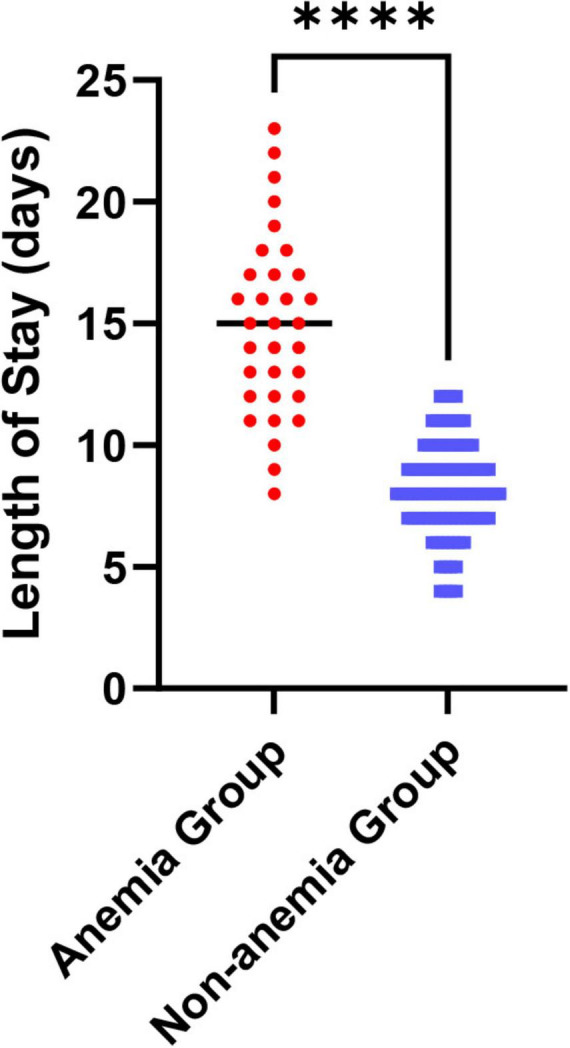
Comparison of average hospital stay between AECOPD patients with and without anemia. Compared with the non-anemia group, ****P < 0.0001.

To clarify the impact of comorbid anemia on the clinical characteristics of AECOPD patients, we compared baseline data between the anemia group (*n* = 32) and the non-anemia group (*n* = 88). As shown in Table 2, significant differences were observed between the two groups in several aspects. The mean age of patients in the anemia group was (73.8 ± 9.5) years, significantly higher than that in the non-anemia group (67.2 ± 8.1) years (*P* = 0.001), indicating that advanced age is a significant risk factor for anemia in AECOPD patients. Regarding nutritional status, the BMI in the anemia group was (19.7 ± 3.5) kg/m^2^, significantly lower than that in the non-anemia group (22.3 ± 3.0) kg/m^2^ (*P* < 0.001). Concurrently, the proportion of patients with comorbid hypoalbuminemia was 53.1% (17/32) in the anemia group, significantly higher than the 27.3% (24/88) in the non-anemia group (*P* = 0.007). This collectively suggests that malnutrition is one of the core pathophysiological components of AECOD-related anemia. Regarding clinical outcomes, the mean hospital stay for the anemia group was significantly longer at (13.5 ± 3.7) days, compared to (9.1 ± 2.8) days in the non-anemia group (*P* < 0.001) (Figure 1), directly demonstrating that comorbid anemia increases disease burden and prolongs the recovery process.

**TABLE 2:** Comparison of clinical characteristics between anemia and non-anemia groups in AECOPD patients.

Parameter	Total (*n* = 120)	Anemia group (*n* = 32)	Non-anemia group (*n* = 88)	*P*-value
Age (years, $\bar{x}$ ± s)	69.2 ± 8.9	73.8 ± 9.5	67.2 ± 8.1	**0.001**
Male [n(%)]	82 (68.3%)	20 (62.5%)	62 (70.5%)	0.403
BMI (kg/m^2^, $\bar{x}$ ± s)	21.8 ± 3.4	19.7 ± 3.5	22.3 ± 3.0	**< 0.001**
Hypoalbuminemia [n(%)]	41 (34.2%)	17 (53.1%)	24 (27.3%)	**0.007**
Length of stay (days, $\bar{x}$ ± s)	10.8 ± 3.6	13.5 ± 3.7	9.1 ± 2.8	**< 0.001**

Data are presented as mean ± standard deviation or number (percentage). *P*-value is for comparison between the anemia and non-anemia groups. Bold values indicate statistically significant differences (*P* < 0.05).

### 3.3 Comparison of red blood cell parameters across different lung function grades

A systematic comparison of RBC parameters among patients with different lung function grades revealed clear and patterned trends. As shown in Table 3, the FEV1%pred value, reflecting the severity of airflow limitation, decreased progressively with increasing grade. Simultaneously, several RBC parameters showed regular changes. Hb levels decreased significantly with worsening lung function, declining from (139.2 ± 9.8) g/L in Grade I to (107.5 ± 10.2) g/L in Grade IV (*P* < 0.001). More revealing were the changes in RDW and MCH: RDW increased consistently with disease severity, rising from (13.5 ± 0.9)% in Grade I to (17.2 ± 1.3)% in Grade IV (*P* < 0.001); conversely, MCH showed a declining trend, decreasing from (29.8 ± 1.7) pg in Grade I to (24.3 ± 2.1) pg in Grade IV (*P* < 0.001). MCV also demonstrated a similar decreasing trend (*P* < 0.001).

**TABLE 3:** Comparison of red blood cell parameters in AECOPD patients by pulmonary function grade (*n* = 120).

Parameter	Grade I (*n* = 22)	Grade II (*n* = 45)	Grade III (*n* = 38)	Grade IV (*n* = 15)	*P*-value
Hb (g/L)	139.2 ± 9.8	129.5 ± 10.5	114.8 ± 11.1	107.5 ± 10.2	**< 0.001**
RDW (%)	13.5 ± 0.9	14.3 ± 1.1	15.9 ± 1.2	17.2 ± 1.3	**< 0.001**
MCH (pg)	29.8 ± 1.7	28.5 ± 2.0	25.9 ± 2.3	24.3 ± 2.1	**< 0.001**
MCV (fL)	91.8 ± 4.8	89.5 ± 5.5	84.9 ± 6.9	80.5 ± 7.2	**< 0.001**

Data are presented as mean ± standard deviation. *P*-value is for comparison among different pulmonary function grades. Hb, hemoglobin; RDW, red cell distribution width; MCH, mean corpuscular hemoglobin; MCV, mean corpuscular volume. Bold values indicate statistically significant differences (*P* < 0.05).

**FIGURE 2:**
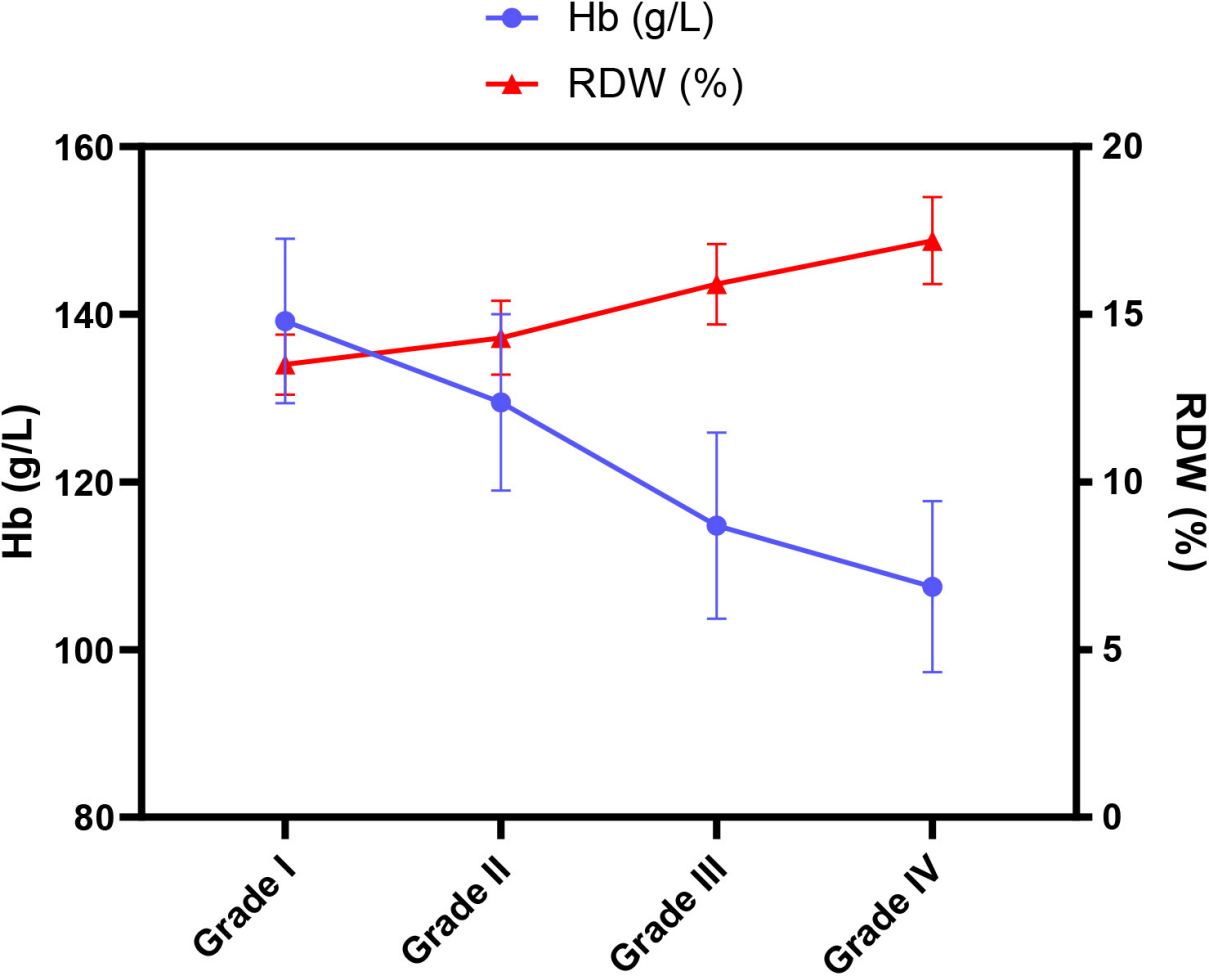
Trends of hemoglobin and red cell distribution width across different pulmonary function grades in AECOPD patients. *P*-values for both Hb and RDW across different grades were < 0.001.

To more intuitively display the dynamic changes of Hb and RDW with disease severity, we plotted a trend graph (Figure 2). This graph clearly shows the line representing Hb declining markedly with worsening lung function grade, while the line representing RDW shows a steep upward trend, forming a distinct scissor-shaped divergence. These data and the graph collectively indicate that in AECOPD patients with poorer lung function, not only is the prevalence of anemia higher, but the morphology of RBCs and Hb synthesis are also significantly altered, manifested as increased RBC size heterogeneity (elevated RDW) and insufficient Hb synthesis (decreased MCH, MCV).

### 3.4 Distribution of anemia types in AECOPD patients

**FIGURE 3:**
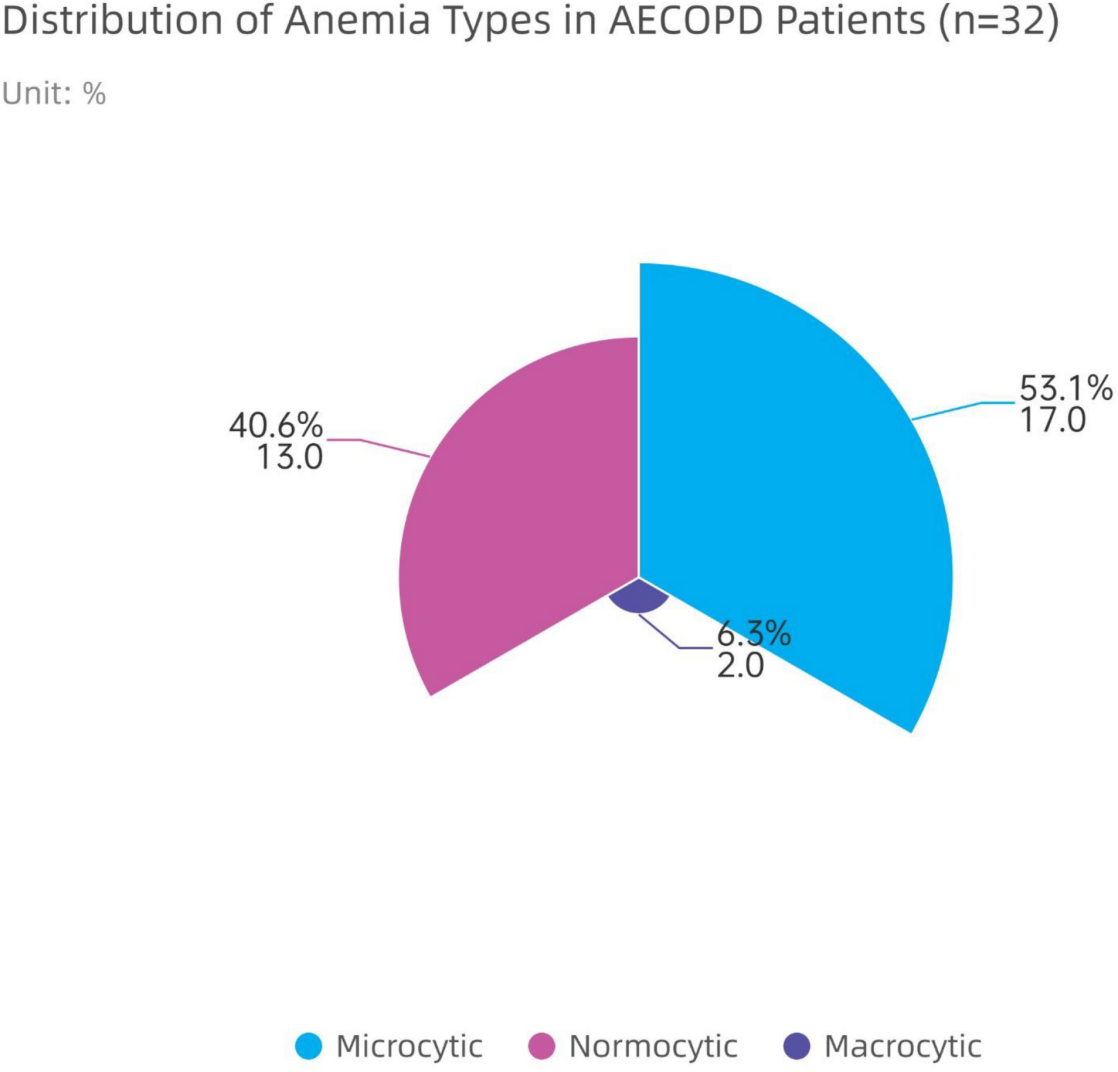
Distribution of anemia types in AECOPD patients with anemia. Based on MCV classification of 32 anemic patients, microcytic anemia accounted for the highest proportion (53.1%).

Preliminary morphological classification of the 32 anemic patients based on MCV revealed the distribution shown in Figure 3. Microcytic anemia (MCV < 80 fL) was the most common, with 17 cases (53.1%); normocytic anemia (80 fL ≤ MCV ≤ 100 fL) followed, with 13 cases (40.6%); macrocytic anemia (MCV > 100 fL) was the least common, with only 2 cases (6.3%). This indicates that anemia comorbid with AECOPD is predominantly microcytic hypochromic anemia, which is compatible with the involvement of iron metabolism disorders (including absolute iron deficiency and functional iron deficiency related to chronic disease) in this type of anemia. However, in the absence of direct iron metabolism indices, this interpretation remains hypothetical and we cannot distinguish between absolute and functional iron deficiency in this cohort.

### 3.5 Correlation analysis between red blood cell parameters and FEV1%pred

**FIGURE 4:**
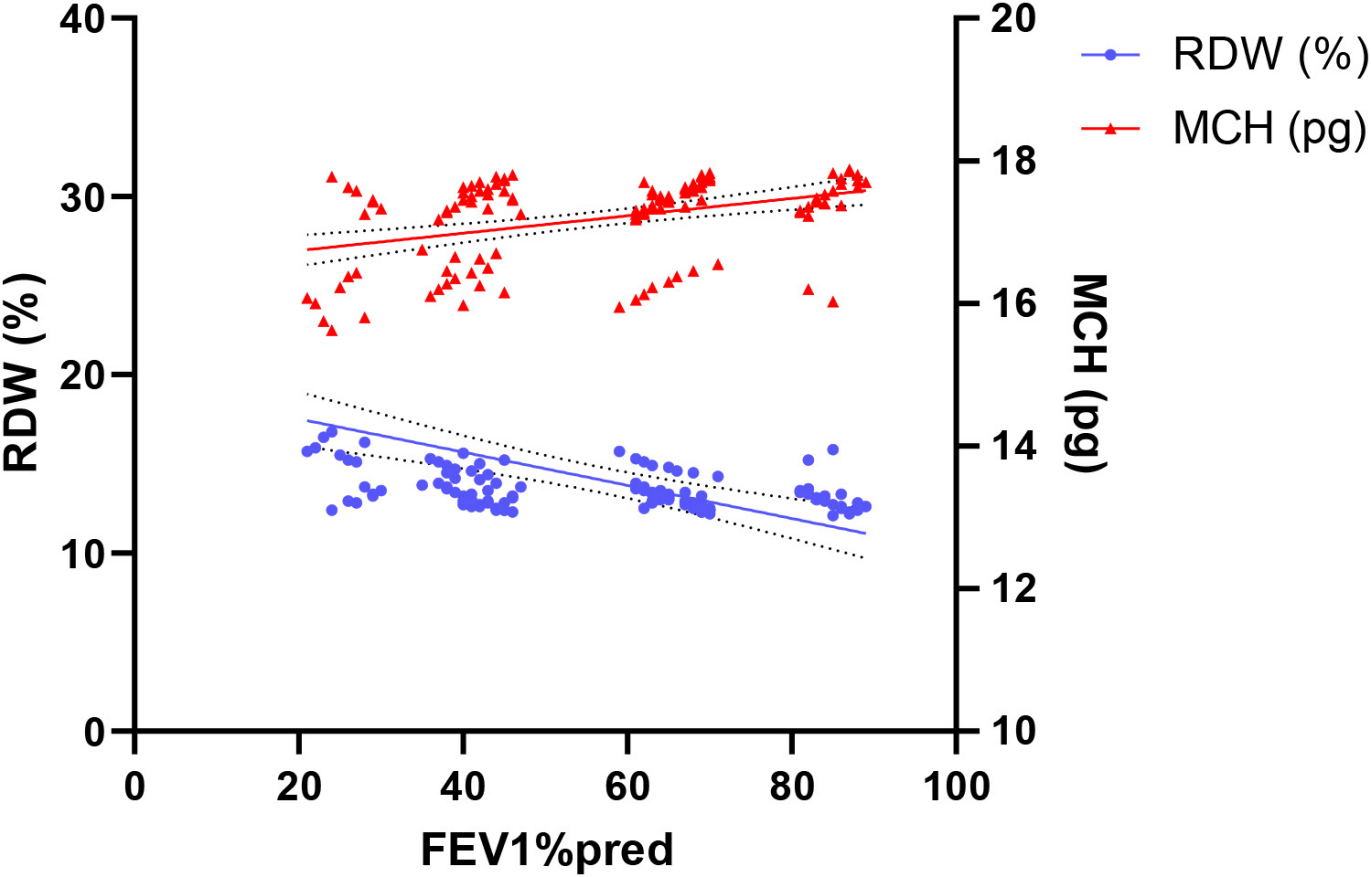
Correlation analysis of RDW and MCH with FEV1% predicted. RDW showed a significant negative correlation with FEV1%pred, while MCH showed a significant positive correlation (both *P* < 0.001).

To further quantify the linear relationship between RBC parameters and lung function, we performed correlation analysis. Pearson correlation analysis results (Figure 4) showed a significant negative correlation between RDW and FEV1%pred values (*r* = –0.418, *P* < 0.001). Conversely, a significant positive correlation existed between MCH and FEV1%pred values (*r* = 0.391, *P* < 0.001). That is, poorer lung function was associated with lower MCH values. These correlation results, from the perspective of continuous variables, reaffirm the conclusions from the earlier group comparisons and directly link RDW and MCH to the degree of lung function impairment.

### 3.6 Predictive value of RDW and MCH for severe AECOPD

**FIGURE 5:**
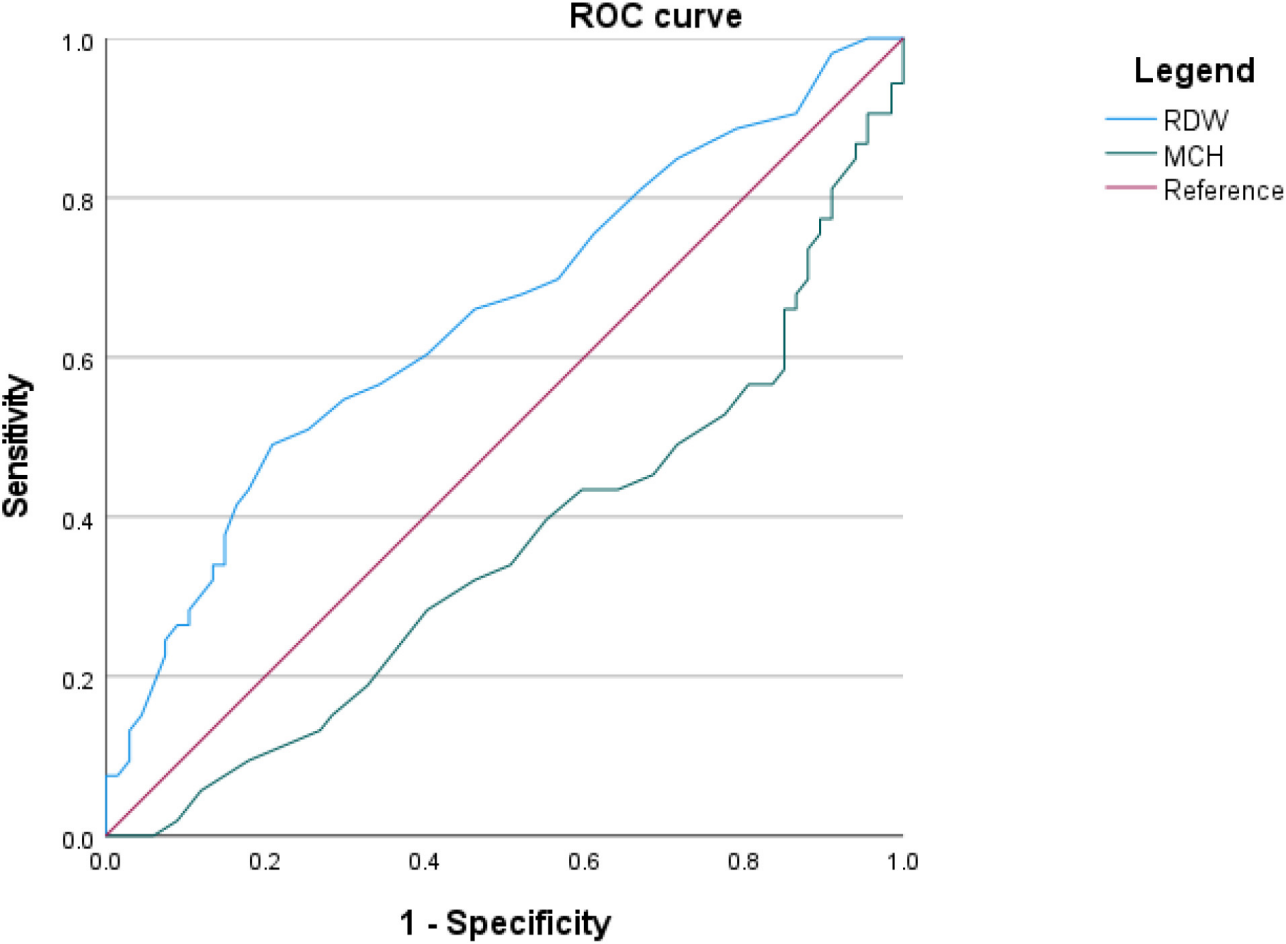
ROC curves of RDW and MCH for predicting severe AECOPD. Receiver operating characteristic (ROC) curves of RDW and MCH for predicting severe AECOPD (GOLD Grades III–IV). For exact AUC values and 95% confidence intervals, please refer to the results section.

To evaluate the ability of RDW and MCH to discriminate severe AECOPD, we performed ROC curve analysis. The results (Figure 5) showed that the area under the curve for RDW in predicting severe AECOPD (Grades III-IV) was 0.652 (95% CI: 0.552–0.752). The AUC for MCH was 0.642 (95% CI: 0.541–0.742). This indicates that both RDW and MCH possess only fair predictive value for identifying severe AECOPD patients, providing modest discrimination when used alone.

### 3.7 Multivariate logistic regression analysis of AECOPD lung function severity

To investigate independent factors influencing the severity of lung function in AECOPD patients, we used lung function grade (defining Grades I-II as 0 and Grades III-IV as 1) as the dependent variable and included variables statistically significant in univariate analysis (age, BMI, Hb, RDW, MCH) in a multivariate binary logistic regression model. Multivariate logistic regression analysis based on standardized variables (Table 4) revealed that, after adjusting for the effects of age, BMI, and MCH, decreased Hb (OR = 0.294, 95% CI: 0.142–0.608) and increased RDW (OR = 2.880, 95% CI: 1.370–6.054) were independently associated with severely impaired lung function (Grades III–IV) in AECOPD patients (*P* < 0.01), and these associations should be regarded as exploratory rather than causal (*P* < 0.01).

**TABLE 4:** Multivariate logistic regression analysis of risk factors for severe pulmonary function in AECOPD patients.

Variable	B	Wald	*P*-value	OR	95% CI
Age	0.512	1.752	0.186	1.669	0.782–3.560
BMI	-0.721	3.654	0.056	0.486	0.232–1.019
Hb (g/L)	-1.225	10.894	0.001	0.294	0.142–0.608
RDW (%)	1.058	7.815	0.005	2.880	1.370–6.054
MCH (pg)	-0.791	3.214	0.073	0.453	0.191–1.074

The dependent variable: pulmonary function severity (0 = Grade I–II, 1 = Grade III–IV). Continuous independent variables were standardized (Z-score) prior to analysis.

### 3.8 Survival analysis of patients with different RDW levels

**FIGURE 6:**
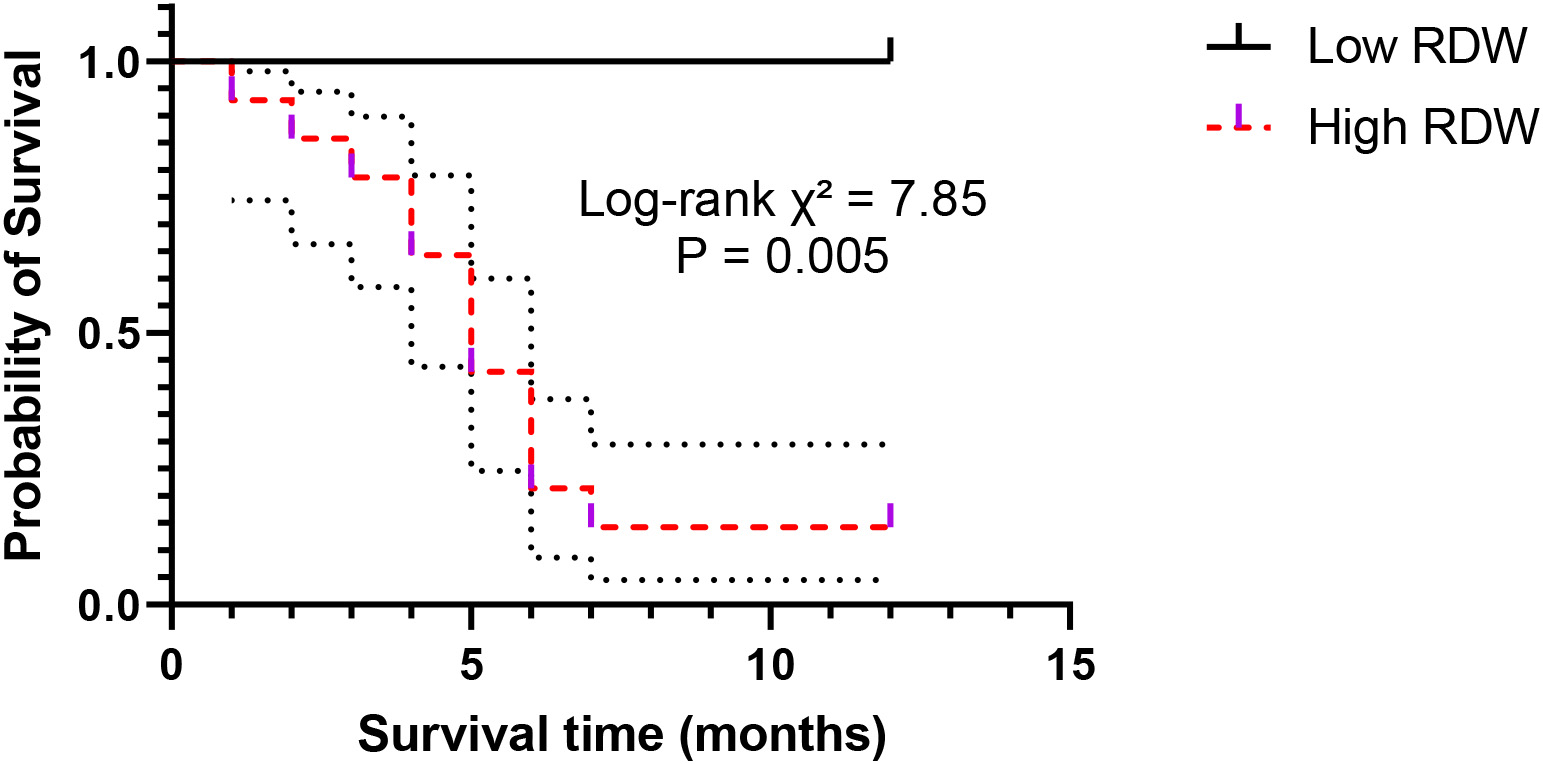
Kaplan-Meier survival curves of AECOPD patients stratified by RDW levels. Patients in the high RDW group had significantly lower cumulative survival than those in the low RDW group (Log-rank test, *P* = 0.005).

To assess the predictive value of RDW for the short-term prognosis of AECOPD patients, we divided patients into a high RDW group (*n* = 60) and a low RDW group (*n* = 60) based on the median RDW value of the entire cohort (14.5%), and followed all patients for a median duration of 12 months. Kaplan-Meier survival curves (Figure 6) showed a significant difference in survival rates between the two groups (Log-rank χ^2^ = 7.85, *P* = 0.005). The cumulative survival rate was significantly lower in the high RDW group compared to the low RDW group, indicating a significantly increased risk of all-cause death within 1 year.

## 4 Discussion

This study, through systematic analysis of clinical data from 120 hospitalized AECOPD patients, reveals a close association between anemia and related RBC parameters with disease severity and prognosis. Our findings indicate that the overall prevalence of anemia in AECOPD patients is as high as 26.7%, and this proportion increases sharply with worsening lung function grades, exceeding half in Grade IV patients (20). This trend coincides with increasing patient age, declining BMI, and decreasing serum albumin levels, collectively depicting a spectrum of “systemic pathophysiological disturbances” related to disease severity (21). This suggests that assessing AECOPD must not be confined solely to the lungs but must integrally include systemic factors such as anemia and nutritional status.

The most pivotal finding of this research lies in establishing a moderate but clinically meaningful association between two often-overlooked routine blood parameters- RDW and MCH-and the degree of lung function impairment in AECOPD. Elevated RDW, indicating increased heterogeneity in RBC volume, is a marker of ineffective erythropoiesis or heightened destruction. Within the chronic inflammatory milieu of AECOPD, persistently high levels of inflammatory cytokines (e.g., IL-6, TNF-α) (22) not only directly suppress the proliferation and differentiation of bone marrow erythroid progenitors but also disrupt iron metabolism homeostasis, leading to upregulated hepcidin levels, impaired intestinal iron absorption (23), and inhibited macrophage iron release, resulting in “functional iron deficiency.” Erythropoiesis under these conditions is inefficient and disordered, directly driving up RDW values Our data showing a significant negative correlation between RDW and FEV1%pred, and its role as an independent factor associated with worse lung function in multivariable regression, aligns with findings from researchers like Sara et al. (24), collectively elevating RDW’s value from a mere anemia differential indicator to a sensitive biomarker reflecting systemic inflammatory burden and oxidative stress levels in AECOPD.

Conversely, our finding that decreased MCH independently correlates with lung function severity holds more direct clinical significance. Low MCH is characteristic of microcytic hypochromic anemia, most commonly associated with iron deficiency. Our analysis of anemia type distribution revealed microcytic anemia accounting for over 50% (25), providing morphological support for the hypothesis that iron metabolism dysfunction may be an important component of AECOPD-related anemia; however, we were unable to verify this mechanism directly because iron metabolism indices were not measured and absolute and functional iron deficiency could not be distinguished. AECOPD patients are prone to absolute or functional iron deficiency due to factors like anorexia from chronic hypoxia and impaired iron utilization due to systemic inflammation (26). Low MCH signifies reduced Hb-carrying capacity per RBC, further compromising blood oxygen-carrying efficiency, exacerbating tissue hypoxia on top of the existing hypoxic physiology, and potentially contributing to lung function deterioration and pulmonary hypertension development (27). While research by Priya et al. (28) identified low MCH as a predictor of poor outcomes in AECOPD patients, our study further anchors this association to the specific dimension of lung function severity, deepening the understanding of its pathophysiological significance.

Abnormalities in these RBC parameters ultimately translate into poorer clinical outcomes. In this study, the anemia group had significantly prolonged hospital stays (29), and the high RDW group showed significantly reduced long-term survival (30), consistent with reports from Maurizio et al. (31). Prolonged hospitalization implies increased healthcare resource consumption and heightened family burden, while survival differences directly impact patient quality of life. Consequently, we advocate for mandatory anemia screening and in-depth interpretation of RBC parameters for AECOPD patients (32), particularly elderly, malnourished, severe cases. When persistent RDW elevation or progressive MCH decline is observed, clinicians should be alert to potential underlying, uncorrected iron metabolism disorders or ongoing inflammatory states, considering further investigations like ferritin and CRP, rather than dismissing them as insignificant comorbidities of chronic disease.

This study has several important limitations. First, it was designed as a single-center retrospective observational cohort of hospitalized AECOPD patients in a tertiary respiratory department. As such, the sample is prone to selection bias, the findings primarily reflect the case-mix and management patterns of this specific hospital, and the results cannot be generalized to broader AECOPD populations without confirmation in multi-center prospective studies. Second, the overall sample size was relatively small (*n* = 120), and the distribution of lung function grades was unbalanced, with only 15 patients in GOLD grade IV. This reduced the statistical power of subgroup and multivariable analyses, particularly for the most severe disease strata and for comparisons between anemia subtypes, so the corresponding estimates should be interpreted with caution and regarded as hypothesis-generating. Third, anemia types were inferred only from MCV and MCH, and systematic testing of iron metabolism indices (such as ferritin, transferrin saturation and serum iron), vitamin B12 and folate was not performed for all anemic patients. Therefore, we could not distinguish absolute from functional iron deficiency, and any mechanistic interpretation that iron metabolism disorders are involved in AECOPD-related anemia remains hypothetical and requires dedicated mechanistic studies. Fourth, because of the limited number of outcome events, only age, BMI and major RBC indices were included in the multivariable logistic regression models. Important potential confounders, including detailed smoking exposure (e.g., pack-years), prior exacerbation history, comorbid cardiovascular disease and systemic corticosteroid use, could not be fully adjusted for, and residual confounding is therefore unavoidable; the observed associations between RBC parameters and lung function severity should be regarded as exploratory rather than definitive. In addition, we used standard WHO hemoglobin cut-offs that do not account for secondary polycythemia in heavy smokers, which may have led to an underestimation of the true prevalence of anemia and should be considered when interpreting the reported anemia rates. Fifth, although C-reactive protein was recorded at baseline, we did not integrate CRP into the multivariable models or conduct formal correlation or mediation analyses. Consequently, the proposed inflammationaanemia–nemiaequently, the proposed ied in the manuscript is supported by existing literature but could not be empirically verified in our dataset. Furthermore, spirometry was only performed after patients had achieved clinical stabilization according to the criteria described in the Methods, which may have preferentially included individuals with relatively milder or improving exacerbations and excluded those who remained too unstable to undergo lung function testing, thereby introducing additional selection bias. Moreover, the median follow-up duration was approximately 12 months, which is relatively short for a chronic disease such as COPD; therefore, our survival analyses reflect only short-term prognosis and cannot address longer-term outcomes. In addition, we used all-cause mortality as the endpoint because reliable attribution of cause-specific death was often difficult in this real-world cohort, which may reduce the specificity of our findings for COPD-related mortality and should be taken into account when interpreting the prognostic implications.

## 5 Conclusion

This study concludes that in AECOPD patients, the prevalence of anemia is related to the severity of lung function impairment. Elevated RDW and decreased MCH are two key and independent RBC parameters reflecting AECOPD severity, respectively revealing core defects in ineffective/heterogeneous erythropoiesis and insufficient Hb synthesis under the disease state. Dynamically monitoring changes in RDW and MCH not only aids in the early identification of high-risk patients with comorbid anemia and iron metabolism disorders but also provides important, economical, and readily accessible laboratory evidence for clinically assessing disease severity, predicting hospitalization duration, and prognosis. Adopting a more comprehensive management strategy for AECOPD, integrating the management of anemia and related RBC parameters into routine assessment and intervention protocols, holds promise as a new breakthrough for improving patient clinical outcomes.

## Data Availability

The original contributions presented in this study are included in this article/supplementary material, further inquiries can be directed to the corresponding author.
